# Latissimus Dorsi Flap Revisited: Coverage of Large Chest Wall Defects Following Mastectomy for Locally Advanced Breast Carcinoma and Angiosarcoma

**DOI:** 10.7759/cureus.53759

**Published:** 2024-02-07

**Authors:** Goran A Ahmed, Rabiya Aseem, Hisham Osman

**Affiliations:** 1 Breast Surgery, Frimley Health NHS Foundation Trust, Frimley, GBR; 2 General Surgery, Royal Surrey County Hospital, Guildford, GBR; 3 Health Economics, University of Surrey, Guildford, GBR

**Keywords:** chest wall surgery, mastectomy, oncoplastic breast surgery, latissimus dorsi myocutaneous flap, secondary breast angiosarcoma, locally advanced breast cancer

## Abstract

Background: The latissimus dorsi myocutaneous flap (LDMF) remains a significant tool in the armamentarium of the oncoplastic breast surgeon. Despite declining popularity for reconstruction, owing to the increasing use of implants and free flaps, it still has an important role in certain salvage situations and as a flap to cover large defects. We report our experience with the use of LDMF for immediate coverage of large mastectomy defects when options are limited.

Methods: Retrospective series of prospectively collected patient records. Patient and tumour characteristics, length of stay, and post-operative and oncologic outcomes are reported. Patients with angiosarcoma were discussed at tertiary sarcoma centres as per national guidelines. Operations were carried out by oncoplastic breast surgeons. The case series was approved by the institutional information governance department in line with institutional requirements for patient data sharing. All patients provided written consent for photography. Descriptive statistics were used to report findings. Median (IQR) was used for continuous variables.

Results: Six women were included, with a median age of 62.5 years, from December 2019 to October 2022. Follow-up ranged from 15 to 49 months. Median tumour size was 72.5 (16.25) mm. Four patients had locally advanced breast carcinoma (LABC), and two had breast angiosarcoma. The donor site and chest wall defects were closed primarily in all cases. Median length of stay was three nights. All mastectomy wounds healed without issues and any delay to their adjuvant treatment. One patient had a minor latissimus dorsi (LD) donor site wound breakdown managed conservatively. Three patients had adjuvant radiotherapy after surgery. Four patients, one after high-grade angiosarcoma and three after aggressive breast carcinoma, had a locoregional recurrence or distant metastases and succumbed within 20 months of surgery.

Conclusion: The LDMF can be a reliable option for the primary closure of large post-mastectomy wounds. Its use can lead to timely wound healing, allowing patients to undergo adjuvant treatment without delay. However, the overall oncologic outcomes in patients with LABC and angiosarcoma are poor due to the underlying aggressive tumour biology. Long-term outcomes are to be interpreted with caution due to the small number of patients with diverse pathologic features.

## Introduction

The role of the latissimus dorsi myocutaneous flap (LDMF) in autologous breast reconstruction has declined over the last two decades with the wider adoption of muscle-sparing free flap reconstructions like deep inferior epigastric perforator (DIEP) flaps and transverse upper gracilis (TUG) flaps [1,2]. However, its role in salvage surgery cannot be abandoned. These include situations like post-radiotherapy breast reconstruction, salvage surgery after failed implant reconstruction, and patients who are not candidates for abdominal flap reconstructions [3]. In addition, it can be used for covering large wound defects in locally advanced breast cancer (LABC), angiosarcoma (AS), and other large chest wall tumours. In this context, several authors have reported the use of LDMF successfully [4-6].

The latissimus dorsi (LD) muscle is a broad, flat muscle that measures up to 25 x 35 cm, located superficially occupying the mid-lower back. When harvested with a skin paddle, depending on the skin laxity of the individual, skin paddles of 31 x 20 cm have been reported, which can cover a large recipient area defect [7]. The first use of the LD flap by Tansini in 1906 was for resurfacing anterior chest wall deformity following radical mastectomy [8]. The aim of using LDMF in this context, is to achieve primary wound closure and provide soft tissue coverage of the resection area, using a well-vascularised skin and muscle flap. This allows for early recovery, prompt wound healing, and timely delivery of adjuvant treatments like radiotherapy [9]. The alternatives like split-thickness skin grafts, may not take after extensive resection and do not tolerate post-operative radiotherapy [10]. Negative pressure wound therapy takes several weeks and delays the delivery of adjuvant treatment. Flaps are more reliable and allow early healing in these patients with rapidly progressive disease [11].

Locally advanced breast cancer is not a common presentation in developed countries nowadays, thanks to increased patient awareness and the availability of breast screening. When presented, upfront surgery for LABC is now less commonly performed due to the availability of neoadjuvant systemic therapy (NAST). However, some patients are not medically fit to have NAST, refuse chemotherapy, or progress while on chemotherapy. This is a difficult clinical scenario as options are limited and expediting surgery is the only option, which often needs to be extensive to achieve local control. Consequently, soft tissue cover will often require flap harvesting. 

In addition to the mastectomy for LABC, radical surgery in the form of extensive wide excision or mastectomy with wide margins, is the only valid option for breast angiosarcoma. Angiosarcoma (AS) is a rare malignant endothelial cancer of vascular or lymphatic origin representing less than 1% of all breast cancers [12]. It can arise de novo as a primary breast angiosarcoma or, more commonly, secondary to lymphoedema or radiotherapy [13]. With the increased use of breast-conserving therapy (BCT) and radiotherapy after mastectomy, the incidence of secondary AS has increased. It manifests in the skin as dark blue ecchymotic skin lesions with or without ulceration and rarely as a breast parenchymal lesion [14]. The median latent period from breast cancer to secondary AS is six years [13,15,16]. Due to the aggressive nature of breast angiosarcomas, radical mastectomy is traditionally performed to improve survival and reduce recurrence rates [17]. However, such excisions result in large defects that are challenging to cover without using pedicled flaps like LDMF or transverse rectus abdominis myocutaneous (TRAM) flaps [18,19].

Due to the decline in the use of LDMF in breast reconstruction, the new generation of surgeons is not very familiar with its indications, surgical technique, and outcomes. Clearly, even with all the advances in cancer management, these cases still exist and can still present a challenge to the oncoplastic surgeon. In this paper, we aimed to add to the existing literature about the use of LDMF for resurfacing purposes. We retrospectively reviewed our experience with the use of LDMF for the surgical management of LABC and angiosarcoma in a single centre.

## Materials and methods

A retrospective analysis of the prospectively collected database was performed. Patients who underwent LDMF for coverage of post-mastectomy defects at a large district general hospital over three years (December 2019-October 2022) were included. The operations were performed by two oncoplastic breast surgeons. All patients were managed in a multidisciplinary setting, including oncologists, radiologists, pathologists, surgeons, breast cancer specialist nurses and geneticists. Patients with angiosarcoma were discussed at a tertiary centre and their treatment was guided by a specialist sarcoma unit. 

Patient demographics, past medical history, tumour characteristics, surgical details, length of stay, and postoperative and oncologic outcomes were recorded and reported. All patients provided consent for photography. Findings were presented narratively and using descriptive statistics. The median (IQR) was used for continuous variables. The study was registered with the information governance department (registration number IG 1677-23). Institutional review board approval was not required due to a lack of patient-identifiable information. 

Surgical technique

**Figure 1:**
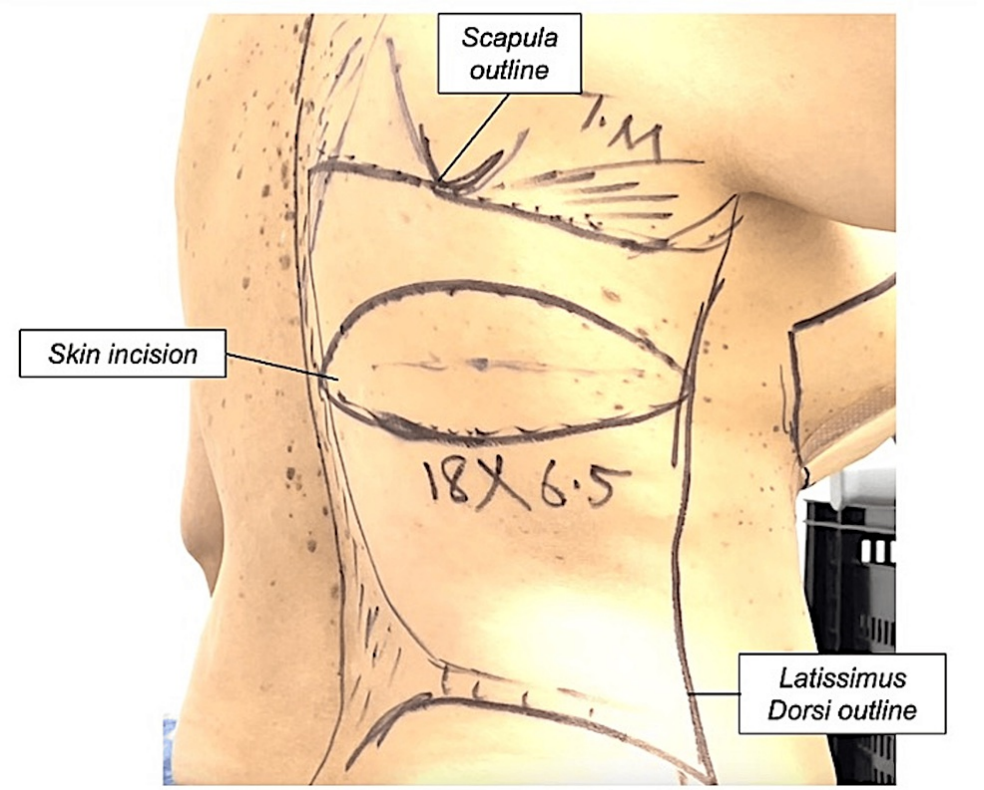
LD flap markings. The LD is outlined and marked. The incision is marked from the axilla or the posterior axillary fold, then inferiorly and medially over the latissimus muscle. LD: latissimus dorsi.

Patients were marked on the day of surgery by the operating surgeon. In the standing position, anteriorly, the midline was marked from the sternal notch to the umbilicus. The extent of mastectomy incision is determined and marked depending on how much skin needs to be excised. On the back, the lateral extent of the LD muscle was marked by drawing a line from the posterior axillary fold down to the iliac crest. The tip of the scapula was marked, and a line was drawn across it to mark the superior border. The posterior border of the iliac crest and midline were marked. The LD muscle lies inside the marked lines. In our series, we designed the elliptical skin paddle horizontally, so the scar lies in the bra line. The width of the skin paddle varied from patient to patient depending on skin elasticity and the patient’s body habitus. We designed the widest possible ellipse by pinching the skin as we wanted the maximum amount of skin to allow for primary skin closure of the mastectomy defect (Figure 1). 

The operation started in the supine position in most patients to perform the mastectomy. In some patients, resection included excising pectoralis major and upper rectus sheath and muscle medially. The wound was dressed with wet packs and a clear film dressing. The patient was then positioned in the lateral decubitus position and prepped and draped in the usual aseptic manner. The skin ellipse was incised, and standard LD muscle harvesting was completed. No attempt was made to do an extended LD, as the aim was not for breast reconstruction but for coverage. The flap was transferred into the mastectomy defect through an axillary tunnel. One or two vacuum drains were used, and the donor site wound was closed in three layers (fascia, subdermal and skin) and dressed. 

The patient was positioned supine again, and the flap was inset. The muscle edges were attached to the rectus fascia, pectoral fascia, and serratus fascia. A vacuum drain was placed in the wound, and the skin was closed. Depending on the shape of the defect, sometimes the skin on either end needed to be detached of the muscle by a few centimetres to allow closure. The wound was dressed making sure to leave the flap visible to monitor vascularity. 

## Results

Six consecutive patients were included over three years (December 2019-October 2022). The median patient age was 62.5 years. The median BMI was 23.9 kg/m^2^. Regarding comorbidities, five patients were American Society of Anaesthesiologists (ASA) class 2, and one was ASA class 3. None of the patients were current smokers. The tumour size was 72.5 mm (IQR: 16.25 mm). The median length of stay (LOS) was three days. All recipient wounds healed without issues. Follow-up ranged from 15 to 49 months. 

Complications were minor and were treated conservatively. One patient developed minimal seroma in the back and one minor donor site wound breakdown that was treated with dressing changes. Table 1 shows patient demographics, tumour characteristics and surgical outcomes. 

**Table 1:** Patient characteristics, histology and surgical outcomes. BMI: Body Mass Index; ASA: American Society of Anaesthesiologists class; IDC: invasive ductal cancer; ILC: invasive lobular cancer; ER: oestrogen receptor; PR: progesterone receptor; HER-2: human epidermal growth factor 2 receptor; WLE: wide local excision; ANC: axillary node clearance; SNB: sentinel node biopsy; RT: radiotherapy; NACT: neoadjuvant chemotherapy; LOS: length of stay; LD: latissimus dorsi.

Case no	Age and gender	BMI	ASA class	Previous breast cancer	Pre-op diagnosis	Neoadjuvant systemic therapy	Surgery	Tumour size	Post-op histology	LOS (days)	Post-op complications
1	53 F	30.4	2	No	Right Breast Triple negative grade 3 IDC	NACT minimal response	Right mastectomy + ANC + LD recon	150 mm	pT4b N3a Mx. One positive node and numerous axillary deposits.	6	Nil
2	83 F	23.9	2	No	Left Breast Grade 3 IDC, T4 breaking through the skin	Declined NACT	Left mastectomy + ANC + LD recon	85 mm	pT4b N2a Mx. One to three positive axillary nodes.	3	Minimal seroma at the donor site.
3	63 F	23.3	2	No	Left Breast Grade 2 ILC, ER, PR 8/8 HER-2 negative	No in view of lobular histology	Left mastectomy + ANC + LD flap recon	65 mm	pT4b, pN3. 17/19 nodes positive for disease.	1	2 cm area of wound breakdown at the donor site, treated conservatively
4	83 F	22.5	3	Yes-WLE and SNB for G2 IDC (RT + hormones)	Left moderately differentiated angiosarcoma	No	Left mastectomy and LD flap recon	75 mm	High-grade angiosarcoma	6	Nil
5	62 F	38	2	Yes-R WLE + ANC 2012 (chemotherapy + RT + hormonal therapy)	Right well-differentiated angiosarcoma	No	Right mastectomy and LD flap recon	65 mm	Low-grade angiosarcoma	3	Nil
6	59 F	23.8	2	No	Right breast metaplastic ca vs sarcoma	No	Right mastectomy and LD flap recon	70 mm	Metaplastic breast cancer PD-L1 negative	3	Nil

Three patients had adjuvant radiotherapy after surgery. Four patients (67%) had local recurrences in the follow-up period. At the time of writing this paper, four patients had passed away. Table 2 shows adjuvant treatment and oncologic outcomes.

**Table 2:** Adjuvant treatment and oncologic outcomes. SCF: supraclavicular fossa.

Case no	Adjuvant treatment	Recurrence	Survival
1	RT to the chest wall and regional nodes PD-L1 inhibitor	Liver metastases a few months later followed by locoregional recurrence	RIP 13 months after surgery
2	RT to the chest wall and regional nodes. Declined palliative chemotherapy	Local recurrence nine months (declined chemo)	RIP 19 months after surgery
3	Declined chemotherapy, adjuvant RT to chest wall and SCF, letrozole for eight years, zoledronic acid	Nil	Alive
4	None	Local recurrence two months postop	RIP six months after surgery
5	None	Nil	Alive
6	Started adjuvant chemotherapy	Five months post op–skin nodules, lung metastases soon after surgery	RIP six months after surgery

Case 1: Resistant inflammatory breast cancer 

**Figure 2:**
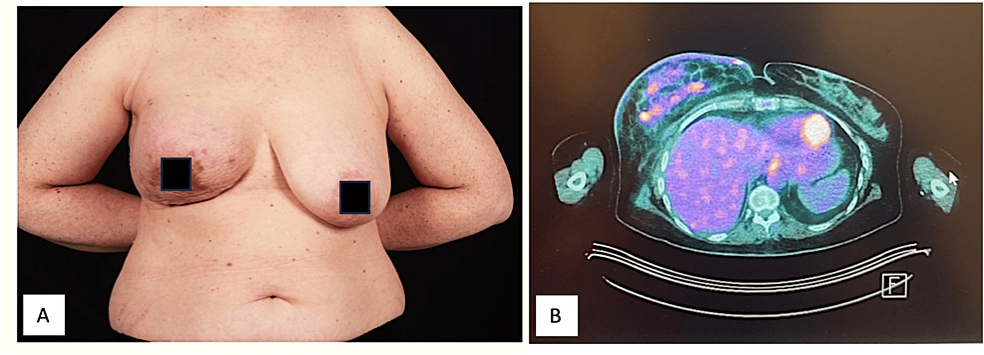
Inflammatory right breast cancer. A: Pre-operative right inflammatory breast cancer after seven cycles of adjuvant chemotherapy. B: PET-CT post-neoadjuvant chemotherapy.

A 53-year-old lady presented with a 2 cm lump behind the right breast areola. The mammogram showed an irregular, asymmetric density in the retro-areolar region, measuring 13 x 14 mm in size with multiple pathological nodes on the ultrasound scan. A core biopsy revealed a G2 invasive ductal cancer (IDC) oestrogen receptor (ER) score of 0/8, a progesterone receptor (PR) score of 0/8, a human epidermal GF receptor (HER-2) was negative, and FNA cytology confirmed a metastatic node. The patient underwent a staging CT scan and bone scan that showed no evidence of metastases but demonstrated subpectoral and supraclavicular suspicious nodes, which were proven to be metastatic on FNAC. The multidisciplinary team (MDT) decision was a referral for neoadjuvant chemotherapy in the form of accelerated EC x four cycles, followed by carboplatin and paclitaxel x four cycles. During treatment, she progressed to have skin redness and oedema in keeping with inflammatory carcinoma. She had a minimal response after the first four cycles and developed severe neuropathy after the seventh cycle. A repeat PET-CT revealed a minor reduction in uptake in the axilla and supraclavicular fossa (SCF) nodes, no distant metastases, but increased intensity of uptake in the breast and skin envelope over a larger area (Figure 2).

The patient then had a right mastectomy and axillary node clearance. Due to the large area of resection, she needed to have LD flap coverage to expedite healing and radiotherapy delivery to the chest wall and regional nodes. On histology, the tumour was 150 mm, and her axillary nodes were all matted together and involved. She healed extremely well without complications and was able to start radiotherapy in less than four weeks. She received 54 Gy over six weeks to the chest wall and regional nodes. Unfortunately, she developed locoregional recurrence and distant metastases shortly afterwards. She was started on palliative chemotherapy and PD-L1 inhibitors but passed away 12 months after surgery.

Case 2: Locally advanced triple-negative breast cancer (TNBC)

**Figure 3:**
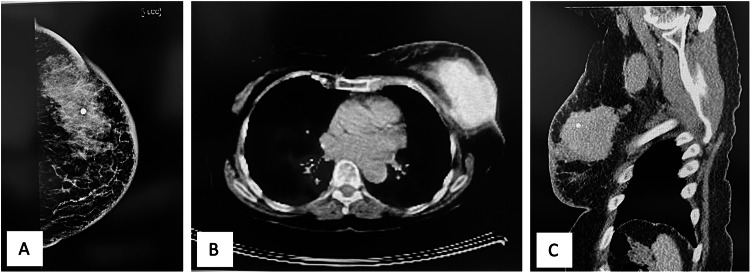
Left breast locally advanced TNBC. A: Left breast mammogram. B: PET-CT showing extensive FDG uptake in the left breast. C: Sagittal view CT scan showing left breast lesion. FDG: fluorodeoxyglucose; TNBC: triple-negative breast cancer.

An 83-year-old lady presented with a 10 cm left breast swelling in the upper outer quadrant involving the skin (T4). Punch biopsy showed G3 IDC ER 0/8, PR 2/8 Allred score and HER-2 negative. The tumour was 85 mm on the mammogram, and there were multiple metastatic ipsilateral axillary nodes confirmed on the core biopsy (Figure 3). The patient declined chemotherapy, so surgery was the only option. She had left mastectomy and axillary node clearance and immediate LDMF for coverage of the large chest wall defect. The patient did not give consent for the publication of her photos. She had an eventful recovery and wound healing with a minor wound breakdown of 2-3 cm on the back wound, but her recipient site healed without issues. Her histology showed a 75 mm cancer with eight involved axillary nodes and thus underwent chest wall and regional wall adjuvant radiotherapy 40 Gy in 15 fractions. She developed recurrence soon afterwards and a distant disease about a year later. She passed away twenty months after surgery.

Case 3: Locally advanced lobular cancer

**Figure 4:**
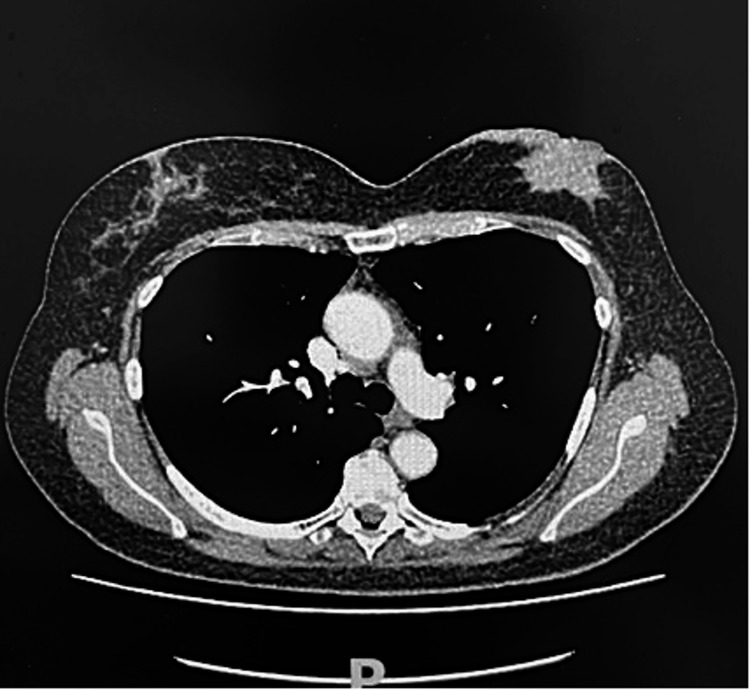
CT images demonstrating fungating locally advanced left-sided breast cancer with skin invasion.

A 63-year-old woman was diagnosed with locally advanced left-sided breast cancer with a gross invasion of the skin and nipple areola complex. Upon initial examination, the tumour was felt to be 10 cm and a core biopsy concluded a G2 invasive lobular cancer of ER 8/8, PR 8/8 and HER-2 negative with involved axillary nodes. The patient did not have a mammogram due to fungating cancer but had a staging CT (Figure 4). Staging and bone scans did not show evidence of distant metastases. 

The patient underwent left mastectomy and axillary node clearance and immediate LDMF for coverage. She had an uneventful recovery. Her recipient wound site healed without major issues, but there was a small 3 cm breakdown of the donor site that healed with conservative measures. The patient had pre- and post-operative photographs taken but declined use for publications. Her histology concluded a 65 mm lobular cancer with the involvement of 17 involved nodes. The patient declined chemotherapy but had adjuvant radiotherapy to the chest wall 40 Gy in 15 fractions and is currently alive on adjuvant letrozole.

Case 4: Secondary angiosarcoma

An 83-year-old lady presented with a painful and diffusely swollen left breast with dark bluish skin nodules. She previously underwent a left-wide local excision (WLE) and sentinel node biopsy (SNB) seven years ago for a 28-mm grade 2 invasive ductal cancer ER 8/8, PR 7/8 and HER2 negative. She had adjuvant radiotherapy and anastrozole. A bilateral mammogram eight months before presentation was normal. A punch biopsy from one of the skin nodules and histology showed moderately differentiated angiosarcoma. Staging CT head, neck, chest, abdomen, and pelvis showed no evidence of distant metastases. Magnetic resonance angiography of the left upper limb was done to ensure the patency of the thoracodorsal pedicle due to previous axillary surgery. This confirmed vessel patency. She was urgently referred to the regional tertiary sarcoma unit and discussed at the sarcoma MDT. It was advised to proceed locally with surgical resection. She was counselled for a left mastectomy and removal of all gross chest wall disease and immediate soft tissue coverage with LDMF. 

**Figure 5:**
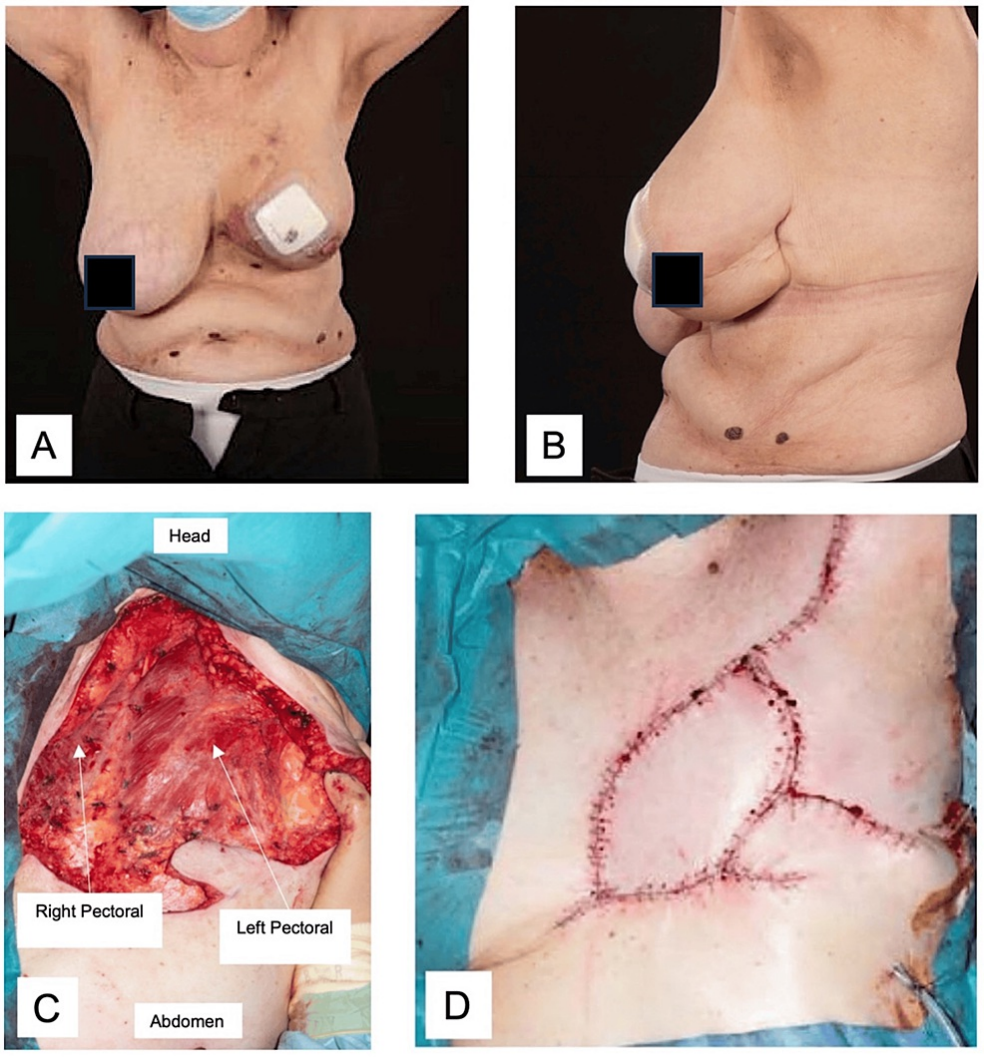
Left breast angiosarcoma. A and B: Pre-operative left-sided angiosarcoma. C: Intraoperative view of defect size. D: Post-operative wound following flap inset and primary closure.

Intraoperatively, all gross disease was removed with macroscopic margins. The extent of resection was large, with an area of ~25 x 25 cm extending to the contralateral breast, as shown in the picture. Primary closure was achieved using skin paddles over LD muscle and local skin advancement flaps (Figure 5). She was discharged on postop day six. All wounds healed without complications. Histology showed extensive high-grade angiosarcoma involving deep resection margin. Radial margins were free of tumour. Unfortunately, she developed local recurrence within two months. She was initially planned for wide local excision of local recurrences, but her disease progressed rapidly locally, and surgery was deemed futile. She was referred to palliative care and passed away six months after surgery. 

Case 5: Secondary angiosarcoma

A 62-year-old lady presented with a few dark nodules on her right breast. She had a background of right breast cancer for nine years before being treated by wide local excision and axillary nodal clearance. She had adjuvant chemoradiotherapy and tamoxifen as part of her initial cancer treatment. A punch biopsy showed a well-differentiated angiosarcoma secondary to previous radiotherapy. Her staging scan ruled out a distant disease. After discussion in the tertiary sarcoma MDT, the decision was to proceed locally with surgery. She underwent the right mastectomy to ensure the removal of all skin nodules with wide margins and immediate LDMF for coverage. Her wounds healed without complications, and she did not require any adjuvant treatment. She is alive and is being followed up with regular clinical reviews and CT scans. The patient had pre-operative photos but declined their use in publications, and there was no abnormality in her scans to share.

Case 6: Metaplastic breast carcinoma 

**Figure 6:**
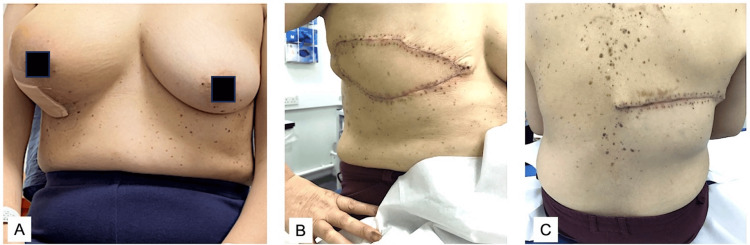
Metaplastic breast carcinoma. A: Pre-operative right-sided metaplastic breast cancer. B and C: Post-operative wound following LD flap reconstruction. LD: latissimus dorsi.

A 59-year-old lady presented with a large painful right breast lump. She had no previous history of breast cancer with a past medical history of coeliac disease. Ultrasound and mammograms showed a 75-mm lobulated mass in the central and lower outer right breast with skin involvement (Figure 6). Axillary nodes were normal clinically and on imaging. She had staging CT-chest abdomen-pelvis (CAP) scans of bilateral small indeterminate lung nodules but no definite area of distant metastases and no axillary lymphadenopathy. A core biopsy was suspicious for a malignant spindle cell tumour but was referred to a tertiary centre for a second opinion. Her second opinion from the histopathologist was also outstanding. In the meantime, she presented to the ED with skin breakdown, an increase in tumour size and oozing from her affected breast. A decision was made to expedite her surgery, and after appropriate counselling and assessment by two surgeons (GA and HO), she had surgery two days later. She was listed for a right mastectomy and immediate LDMF for coverage.

At surgery, she had a radical right mastectomy with en bloc resection of most of the pectoralis major, upper right rectus muscle and part of the serratus anterior muscle. The decision was made not to perform axillary surgery due to normal radiology and a clinical exam with a histopathological suspicion of sarcoma. LDMF was harvested with as large a skin paddle as possible to allow primary closure of both donor and recipient sites. She had a smooth hospital course and was discharged on day three. Her wounds healed without any issues, and she was seen weekly for the first few weeks. Her histology showed a 90 mm tumour most likely metaplastic carcinoma, but also suspicious for sarcomatous growth in malignant Phyllodes. Margins were free of tumour. 

On re-staging with a PET-CT scan a month post-operatively, she had multiple lung metastases. She was seen by the oncologists and started adjuvant chemotherapy. She failed to achieve a good response to chemotherapy and unfortunately passed away six months post-surgery. 

## Discussion

The aim of this paper was to share our experience and revisit LDMF use as a salvage tool for the closure of large post-mastectomy defects after LABC and angiosarcoma. There have been a few previous case series, and more literature is needed to guide clinicians, in particular the new generation of oncoplastic surgeons, in these challenging clinical scenarios [5,6].

Our experience confirms the reliability and versatility of LDMF in covering of soft tissue defects with a 100% healing rate in both the donor and recipient areas. The flap has a robust blood supply, which allows for prompt healing in time for any adjuvant treatment required. In our series, none of the patients experienced total or partial flap necrosis. In a recent systematic review of pedicled LD breast reconstruction, Peshel et al. reported a low rate of flap necrosis (partial or total) of 4% [20]. Similarly, in a large cohort of 208 patients, Wattoo et al. reported a flap loss rate of 3.4% [21]. 

In a retrospective case series of LDMF use in upper limb and chest wall reconstruction, Naalla et al. included 12 patients who had LDMF coverage of post-radial mastectomy defects with no major complications. One patient had a partial wound breakdown, which was healed by secondary intention. The size of the wound was the largest in their series and was 26 x 18 cm [9]. In our series, one patient had a defect that was similar in size, but we were able to close it primarily with LDMF and local advancement flaps without healing complications. 

Over the last 50 years, surgery for breast cancer has seen a major de-escalation. Currently, extensive mastectomy, where primary skin closure is not possible, is rarely performed [9,12]. It is reserved for patients with locally advanced breast cancer who fail to respond to neoadjuvant chemotherapy or are not suitable for it. Furthermore, mastectomy with wide margins also seems to be the only viable option for angiosarcoma. In a study about surgical management of AS, comparing “conservative” resections limited to wide excision of gross disease versus “radical” excision including mastectomy and the entire irradiated field, Li et al. found a significantly reduced recurrence rate, lower metastases and improved survival with radical resections [17].

As the incidence of secondary angiosarcoma is increasing, clinicians have to be vigilant of its manifestations and biopsy any suspicious skin lesions that arise some years after treatment for breast cancer [14,22]. Furthermore, surgeons need to be aware of management options. In the UK, all sarcoma patients need to be referred to tertiary sarcoma units for management. In our experience with both angiosarcoma patients, due to pressure on the tertiary centres, after discussion, the advice was to proceed with surgery locally. The new generation of breast surgeons in the UK may not have experience of the radical resection required and LD flap reconstruction and seeking help from senior colleagues or joint operating with sarcoma and plastic surgeons is necessary to optimise patient outcomes [23]. 

Other flaps reported in this setting include transverse rectus abdominis myocutaneous (TRAM) flaps and free flaps. However, these operations usually take longer and have a higher risk of complications and donor site morbidity. LDMF has a low risk of complications and donor site morbidity. The downside is shoulder weakness, which is an acceptable trade-off given the advantage it provides in this setting [9,24].

Oncological outcomes in LABC and high-grade angiosarcoma are poor due to the underlying biology and high risk of locoregional recurrence and haematogenous spread [25]. In a histopathological case series of secondary angiosarcoma after breast cancer, 14 out of 22 patients had local recurrence and nine had metastatic disease at a median of 44 months [26]. Our patients were no different, and despite the use of adjuvant treatment where indicated, a poor outcome was observed, and two-thirds of our series experienced disease progression and died during the follow-up period.

Our series has several limitations. Firstly, the small number of patients included given the uncommon indication of LDMF and the pathological types of cancers treated. Secondly, the diverse pathologic types and variable follow-up, limit making conclusions about the oncologic outcomes. Furthermore, our trust underwent a software change, and all patient information was uploaded onto a new software, which caused the loss of key patient information. This included the size of the surgical defects and LD skin paddle dimensions. Additionally, some patients consented to photography but did not consent to pictures being published; hence, those have not been included in the paper. 

## Conclusions

Despite its declining role in breast reconstruction, the latissimus dorsi myocutaneous flap can provide reliable soft tissue cover after wide mastectomy for local advanced breast carcinoma and angiosarcoma. The operation, recovery and healing are usually straightforward, which is important to avoid delay of adjuvant therapy in this cohort of high-risk patients. However, oncologic outcomes are still poor due to the progressive nature and treatment-resistant disease, although making firm conclusions is difficult due to the heterogeneous cancers included in this series.
